# Plant-parasitic nematode research in the arid desert landscape: a systematic review of challenges and bridging interventions

**DOI:** 10.3389/fpls.2024.1432311

**Published:** 2024-07-22

**Authors:** Ahmed Elhady, Linah Alghanmi, Mahfouz M. M. Abd-Elgawad, Holger Heuer, Maged M. Saad, Heribert Hirt

**Affiliations:** ^1^ DARWIN21, Biological and Environmental Sciences and Engineering Division, King Abdullah University of Science and Technology (KAUST), Thuwal, Saudi Arabia; ^2^ Plant Pathology Department, National Research Centre, Giza, Egypt; ^3^ Institute for Epidemiology and Pathogen Diagnostics, Julius Kühn Institute (JKI) - Federal Research Centre for Cultivated Plants, Braunschweig, Germany

**Keywords:** plant-parasitic nematode research, interventions, MENA region, arid soil development, accelerating progress, resilient management

## Abstract

Plant-parasitic nematode research in the Middle East and North Africa (MENA) region faces significant challenges rooted in a need for proper assembly, diversity, and a unified and purpose-driven framework. This led to exacerbating their detrimental effects on crop production. This systematic review addresses the current situation and challenges that require targeted interventions to sustainably manage plant-parasitic nematodes and reduce their detrimental impact on agriculture production in the MENA region. We analyzed the nematode-related research conducted within the region over the past three decades to assess available resources and promote diverse research approaches beyond basic morphology-focused surveys. We show that crops are attacked by a diverse spectrum of plant-parasitic nematodes that exceed the global economic threshold limits. In particular, *Meloidogyne* species exceed the threshold limit by 8 - 14-fold, with a 100% frequency of occurrence in the collected soil samples, posing a catastrophic threat to crop production and the economy. We highlight detrimental agriculture practices in the MENA region, such as transferring soil from established fields to barren land, which enhances the dissemination of plant-parasitic nematodes, disrupting soil ecology and causing significant agricultural challenges in newly cultivated areas. Looking into the behavior of farmers, raising awareness must be accompanied by available solutions, as more practical alternatives are needed to gain the confidence of the farmers. We propose integrating microbial-based products and soil development practices in hygienic farming as resilient and sustainable solutions for nematode management. Increased emphasis is required to diversify the nematode-related research areas to bridge the gaps and facilitate the transition from fundamental knowledge to practical solutions. A cohesive network of nematologists and collaboration with national and international entities is crucial for exchanging knowledge related to legislation against invasive species.

## 1 Introduction

The MENA region is greatly affected by climate change, worsening existing social and economic issues (Wehrey and Fawal, 2022). With limited arable land and water, the MENA region heavily depends on international food markets, making it one of the largest net importers globally (Le Mouël et al., 2023). This reliance on imports, coupled with uncertainties in supply and demand, aggravates the situation and raises concerns about food security and dietary and nutritional problems (FAO et al., 2021), especially after the COVID-19 pandemic (Mandour, 2021). In 2011, the import dependency in the MENA region was estimated to be up to 40% of their domestic food needs (Marty et al., 2018), but climate change and existing consumer patterns in the MENA region will lead to a rising import dependency on food, reaching 50% to 70% of domestic needs by 2050 (Le Mouël et al., 2023), if decisive actions were not taken. The temperature has increased over the past three decades to 1.5°C in the Middle East (Agoumi et al., 2022) and is projected to rise to 3°C by 2050 (Lelieveld et al., 2016). Subsequently, the MENA region, especially in North Africa, Syria, and Iraq, has seen an up to 10% decrease in rainfall in recent decades (Karami, 2019; ICRC, 2021).

Agricultural practices and rural development urgently require updating to address climate variability and its harmful impacts on land and water resources (Ahsan et al., 2021). Only a tiny portion of the MENA region’s land is suitable for agriculture, with only 5% being arable (OECD/FAO, 2018). Approximately 40% of the cropped area relies on irrigation, while approximately 4% of the land is suitable for rain-fed cereal cultivation (OECD/FAO, 2018). The soil used for farming is severely degraded by up to 40-70% (Oxford Analytica, 2022), reducing agricultural productivity by 30 to 35% compared to their potential (OECD/FAO, 2018). Compared to other regions, the MENA region exhibits low agricultural productivity (FAO and IFPRI, 2018). This is primarily due to the high proportion of arable land used for low-yield crops with traditional, not advanced agricultural practices and the low productivity of desert pastures. Small-size farming and land segmentation limit the introduction of new technologies and investments (Bertini and Zouache, 2021). Water scarcity is also a pressing issue, with two-thirds of the countries depleting groundwater at rates exceeding renewable freshwater resources (Odhiambo, 2017). Thus, MENA countries are prone to frequent droughts, which lead to yield instability and potentially cause a decline of up to 21% in agricultural production by the end of the century (Hejazi et al., 2023).

Such challenges in the MENA region are exacerbated by various phytopathogens, leading to quantitative and qualitative deterioration in crop yields. Spontaneous introduction of alien pests to the MENA region could further aggravate the matter. For instance, in 2016, the fall armyworm, *Spodoptera frugiperda*, emerged as a significant threat, inflicting severe damage on over 80 to 353 distinct crops, often resulting in near-complete yield loss (Paudel Timilsena et al., 2022). The tomato leafminer, *Tuta absoluta*, has also been identified as a cause of yield reductions ranging from 11% to 43% (CABI, 2019). Similarly, the red palm weevil (*Rhynchophorus ferrugineus*) has been linked to an estimated annual economic loss of €483 million (Yaseen, 2019). Moving beyond these concerns, the MENA region faces a formidable threat of expansive desert locust swarms, posing a substantial risk to food security across the region (Malek, 2021). Concurrently, the persistent emergence of new and aggressive races of wheat stripe rust (*Puccinia striiformis* f. sp. tritici) further exacerbates the constraints on wheat production in the same region (Esmail et al., 2021).

Plant nematology is a relatively young topic compared to other fields of plant pathology (Wang et al., 2008) with the initial focus on taxonomy, but research quickly shifted toward understanding the biology, physiology, interaction with host plants and ecological behavior (Eves-van den Akker et al., 2021). Phytonematology research in the tropical regions was rare and most tropical nematode-related field experiments and identifications were conducted in the USA and European laboratories (Waele and Elsen, 2007). Recently, however, the scientific community’s interest in plant-parasitic nematodes has been growing in the MENA region due to the significant damage and losses in crop production (Abd-Elgawad and Askary, 2015) but also due to the contribution of free-living nematodes to nutrient cycling and soil health (Laasli et al., 2022). In this review, we aim to analyze the current situation of the nematode-related research proposing a roadmap for future research and agricultural applications in the MENA region.

## 2 Materials and methods

### 2.1 Selection criteria for plant-parasitic nematode studies in the MENA region

We analyzed the plant-parasitic nematode-related research conducted within the region over the past three decades to identify the dominant plant-parasitic nematodes, their incidence ranges, and associated damaged crops. We included 58 peer-reviewed studies available in English that were conducted between 1994 and 2023. Articles published in Arabic were excluded due to their limited accessibility. The literature search and collection process occurred between June 2023 and December 2023. Our screening process covered studies from 13 countries: Algeria, Egypt, Iraq, Jordan, Libya, Morocco, Northern Iran, Oman, Palestine, Saudi Arabia, Sudan, Syria, and Tunisia. We recorded the frequency of occurrence (FO%) of nematode species, whether as individual values or ranges, to facilitate analysis. We summarized the minimum and maximum nematode population density for each species and their respective host crops, focusing on those with at least a 5% frequency occurrence (**Supplementary Table S1**). We used the recently available reports from various agricultural organizations to address the current production of economic crops. For instance, the citrus production in Syria, Tunisia, Morocco, and Saudi Arabia was referred to the organizational reports from the Food and Agriculture Organization (FAO, 2015, 2021) and the World Data Atlas (2021). However, we extracted production values in tons for olive production from the International Olive Council (ICO, 2022) report. We also referenced the reports from the FAO (2023) and the USDA Foreign Agricultural Service (2022) to gather insights into the prevailing situation of cereal production within the region.

### 2.2 Data normalization and visualization

Normalization of the nematode’s population densities was implemented to account for differences in soil sampling sizes and standardization of the data in the various studies ensuring that all data were represented consistently with a 100 cm3 soil sample size. It is known that the damage threshold limit is not a static parameter but is instead influenced by many variables such as plant species, cultivar, geographical location, soil composition, and other environmental factors that determine the point at which nematode populations trigger significant yield losses and necessitate immediate intervention. Hence, we have employed the prevailing threshold limits associated with each genus or species to assess the present status of nematode populations within the MENA region compared to the available information in it or other regions (**Supplementary Table S2**). Bullet graphs depict bar variations and incorporate global economic damage threshold limits as contextual backgrounds to visualize the data. This enabled a clear assessment of whether the abundance range of each nematode species fell within or exceeded the threshold limits.

### 2.3 Screening of nematode-related challenges in the MENA region

We also screened the literature to outline the challenges associated with nematode management in the MENA region, focusing on agricultural practices and regulatory and legislative frameworks. For instance, we screened agricultural practices aiming to reclaim desert land by farmers, which led to the dissemination of plant-parasitic nematodes. Furthermore, we highlighted the need for more legislative policies for nematode management in the MENA region.

## 3 Results

### 3.1 A diverse spectrum of plant-parasitic nematodes exceed the global economic threshold limits in the MENA region

**Figure 1:**
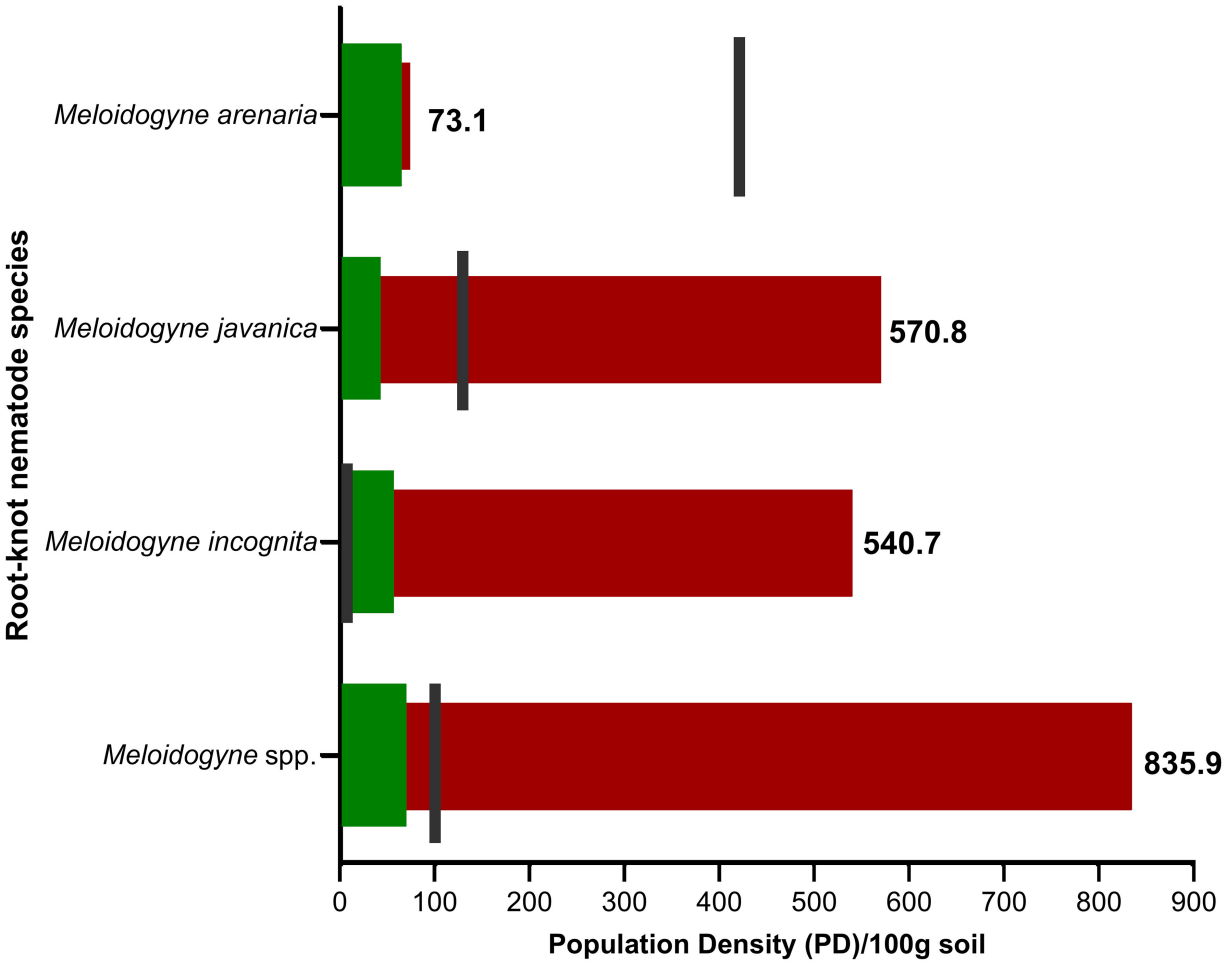
A bullet graph shows the population density (PD) status of *Meloidogyne* species (J2) per 100 grams of soil in the MENA region during the last three decades. The green columns represent the lowest population density values, while the red columns represent the highest values. The black lines represent each species’ known or most widespread damage threshold limit (Todd and Jardine, 1993; Korayem et al., 2012; Xing and Westphal, 2012; Nadeem et al., 2023).

Screening the plant-parasitic nematode research carried out in the MENA region revealed a diversity of plant-parasitic nematodes across the MENA region encompassing numerous genera, including *Aglenchus*, *Aphelenchus*, *Aphelenchoides*, *Bitylenchus*, *Coslenchus*, *Criconema, Ditylenchus*, *Hoplolaimus*, *Helicotylenchus*, *Hemicriconemoides*, *Heterodera*, *Longidorus*, *Malenchus*, *Meloidogyne*, *Merlinius*, *Mesocriconema*, *Panagerllus*, *Pratylenchus*, *Radophulus*, *Rotylenchulus*, *Tylenchorhynchus*, *Tylenchulus*, and *Xiphinema*. Of particular significance, the *Meloidogyne* genus dominates the nematode population in the region. Three primary species have been observed across the MENA region survey studies: *M. incognita*, *M. javanica*, and *M. arenaria*. Generally, the population density of *Meloidogyne* nematodes exceeds the known damage threshold limit by a factor of 8- 14-fold with 100% frequency of occurrence in the collected soil samples. In some instances, exceptionally high densities of *Meloidogyne* spp. were observed. For example, in the reclaimed sandy soil at SEKEM farm in Egypt, the density was quantified at a surprising rate of 17030 second-stage juveniles (J2s)/200g soil (Adam et al., 2013). Similarly, in Saudi Arabia’s Taif region, the density reached 3600 J2s/250g soil (Nour El-Deen et al., 2015). When considering the damage threshold limit for *M. incognita*, as predicted for watermelon cultivation in sandy soil (Xing and Westphal, 2012), the population density in the MENA region exceeds this threshold by 150-fold, with occurrence frequencies ranging from 18 - 73%. We also noted that *M. javanica* exceeds the damage threshold limit by a factor of 4 times, while *M. arenaria* remains within safe limits, not exceeding the threshold (**Figure 1**).

**Figure 2:**
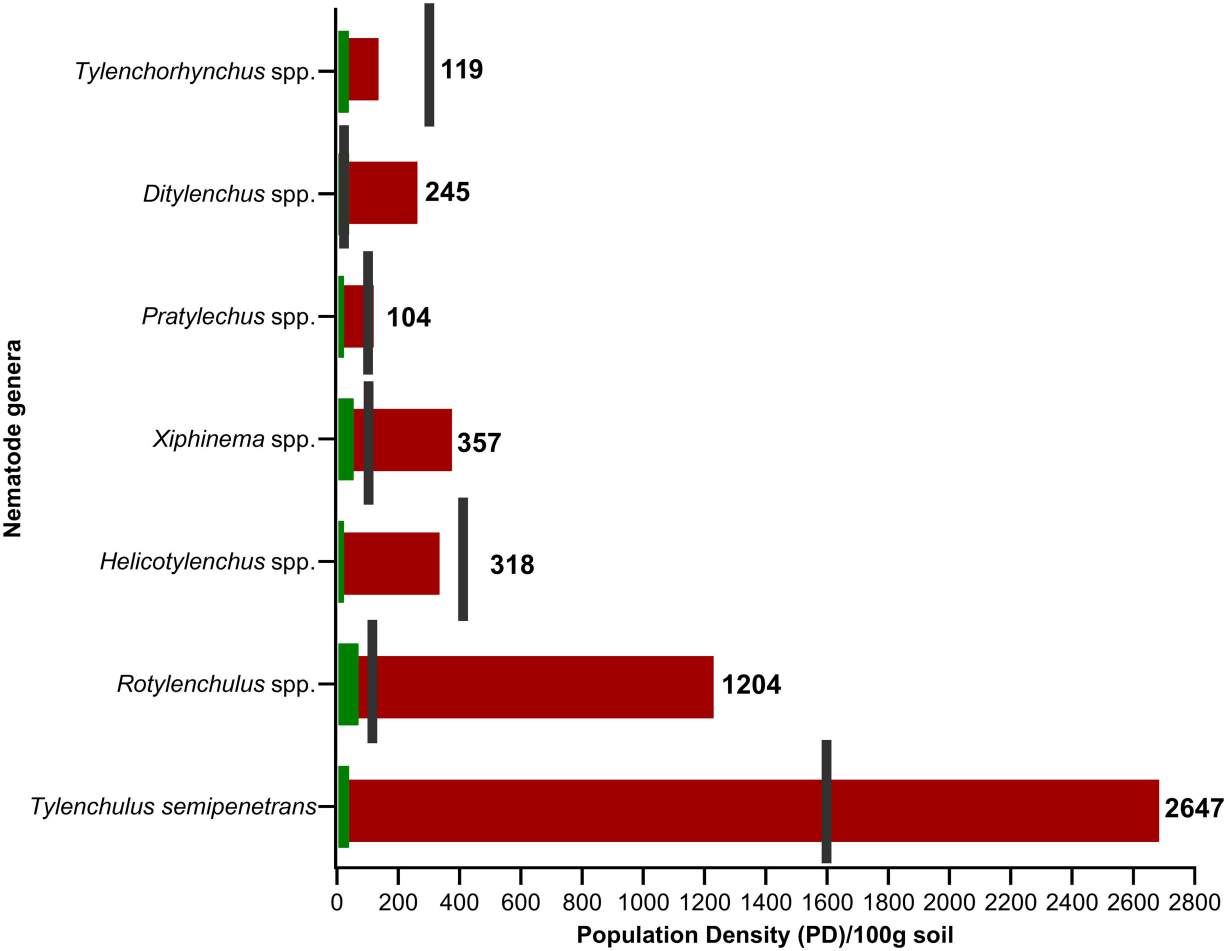
A bullet graph shows the population density (PD) status of different plant-parasitic nematode genera (juveniles and adults) per 100 grams of soil in the MENA region during the last three decades. The green columns represent the lowest population density values, while the red columns represent the highest values. The black line represents each species’ known or most widespread damage threshold limit (Couch, 1995; Showmaker et al., 2011; Fleming et al., 2016; Teklu et al., 2016; Brinkman and Teklu, 2021).

On the other hand, *Tylenchulus semipenetrans* pose a significant threat to citrus production, especially in Egypt and Morocco, with occurrence frequencies ranging from 6.6 - 100%. Using the estimated damage threshold limit (Bozbuga et al., 2023), the abundance of *T. semipenetrans* in the MENA region transcended this limit 1.65 times. In Egypt, extremely high population densities ranging from 1,906 to 9,600 J2s per 200g of soil were observed in citrus fields (Bakr et al., 2011). *Rotylenchus* spp. was detected in exceptionally high numbers, reaching 12600 individuals per 250cm³ of soil on date palm (*Phoenix dactylifera* L.) in Oman (Mani et al., 2005). Their population density in the MENA region also exceeds the damage threshold limit suggested (Showmaker et al., 2011) by 12-fold. Both *Xiphinema* spp. and *Ditylenchus* spp. are present with low occurrence frequencies but high densities that greatly exceed the damage threshold limits in the areas where they were detected, including Egypt, Saudi Arabia, Morocco, and Sudan. For example, the population density of *Ditylenchus* spp. exceeds the damage threshold limit suggested (Brinkman and Teklu, 2021) by 19-122 times. Meanwhile, *Xiphinema* spp., whose damage is associated more with their ability to transmit viruses, exceeds the damage threshold limit (Couch, 1995) by 3.56 folds. Other species, such as *Helicotylenchus* spp., *Pratylenchus* spp., and *Tylenchorhynchus* spp., are found within a safe zone, with population densities frequently lower than their respective damage threshold limits (**Figure 2**). Cyst nematodes, specifically *Heterodera* spp., have been identified within the MENA region. Among the various species, *Heterodera avenae*, the cereal cyst nematode, emerges as the predominant species with a frequency of occurrence ranging from 8-92% in wheat fields. In Ismailia, Egypt, there was a notable incidence of *H. avenae*, with 35 and 46 cysts per 100g of soil (Baklawa et al., 2015; Korayem and Mohamed, 2018).

On the other hand, the date palm *Phoenix dactylifera* L. is also an economic crop in the Middle East. Analysis of root and soil samples revealed the prevalence of different plant-parasitic nematode species like *Meloidogyne* spp., *Pratylenchus* spp., *Criconemoides* spp., *Helicotylenchus* spp. that seriously affect yield (Youssef, 2014). The early infection of date palm seedlings with *M. incognita* reduced the shoot dry weight by 21-33% in a cultivar-dependent manner (Eissa et al., 1998).

#### 3.1.1 *Meloidogyne* spp. represent a critical challenge to key crops in the MENA region

**Figure 3:**
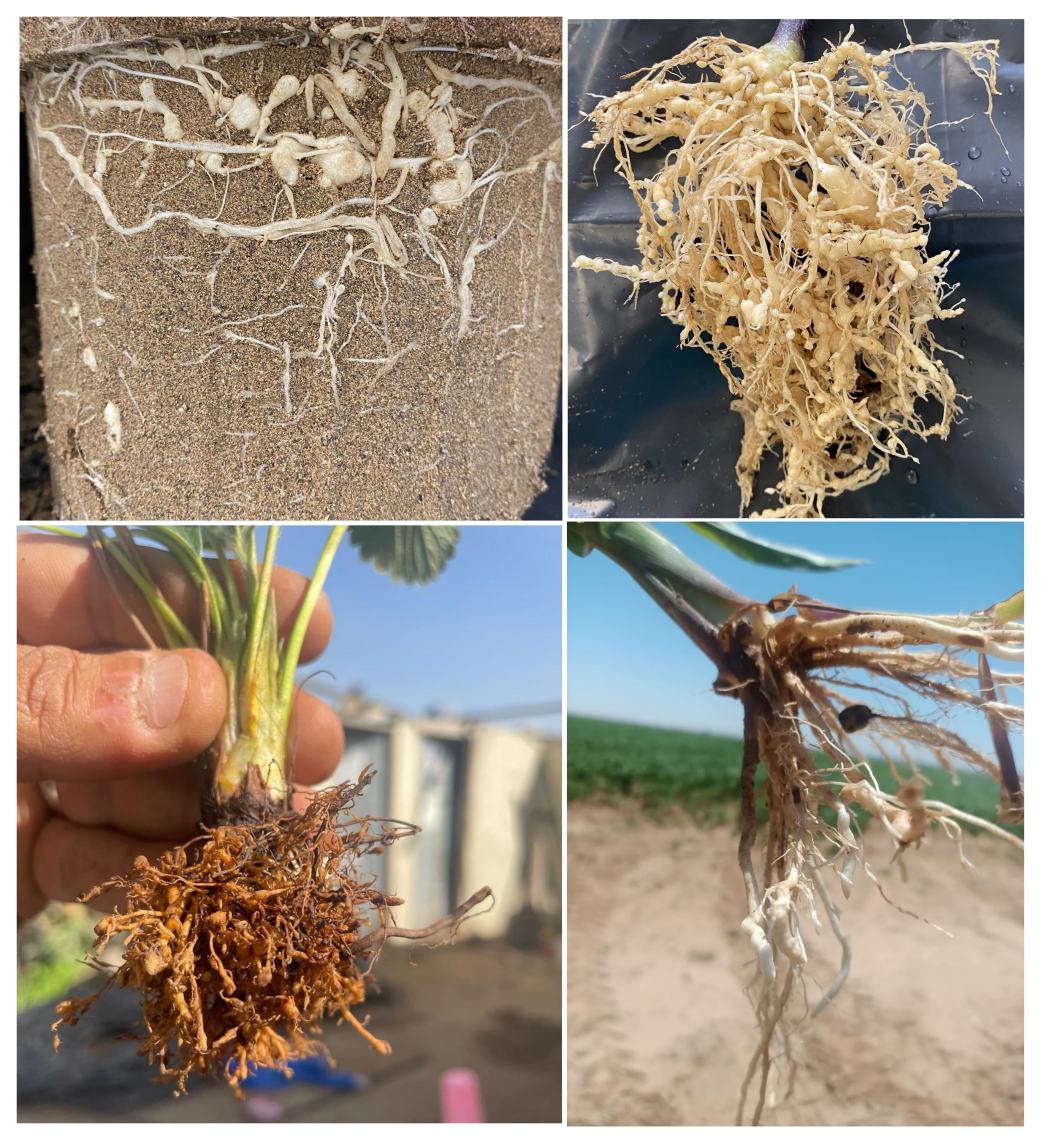
Infection of different crops with *Meloidogyne incognita* shows intensive damage and galls that cover the whole root system. The top left and right pictures represent tomato plants of the “Moneymaker” cultivar with *Meloidogyne incognita* at greenhouse conditions. The bottom left shows the galls on the roots of strawberry seedlings (on the left) and maize (on the right) infected with *Meloidogyne* in a field in Egypt.

*Meloidogyne* spp. poses a significant threat to vegetables, especially for tomato and strawberry producers in the MENA region (**Figure 3**). Tomato is one of the most common vegetables that grow in different seasons across the year in the MENA region. The average tomato yield was estimated at 37.8 tonnes per ha between 2020-2016 (FAO, 2018). In Egypt, *Meloidogyne* spp. were the most prevalent nematodes associated with tomatoes, found in 62.5% of occurrences (Ibrahim et al., 2010), while they caused about 60% loss of the tomato yield in Tunisia (Horrigue-Raouani, 2003). In Morocco, five populations of *M. incognita* were identified and the population density ranged between 59 to 121 per 100 cubic centimeters (Janati et al., 2018). On the other hand, Egypt and Morocco constitute the largest strawberry industry in Africa and the Middle East. Specifically, annual production averaged 394,500 tons in Egypt, 138,400 thousand tons in Morocco, 9,700 tons in Tunisia, and 8,000 tons in Jordan, Lebanon, and Palestine collectively between 2015 and 2017 (Hancock, 2020). Only Egypt contributes 5% to the global strawberry production market. In fields consistently cultivated with strawberries (**Figure 3**), *Meloidogyne* spp. infested 96% of the newly reclaimed fields sampled (Bakr et al., 2011). In Egypt, the frequency of occurrence of *Meloidogyne* spp. in strawberry fields varies significantly across different regions and ranges between 30-41%, with an average reaching 704.5 juveniles/kg soil (El-Habashy, 2010). Meanwhile, maize is an essential grain crop for livestock feeding in the MENA region. Despite the expansion of maize cultivation, there remains a significant demand for imported maize, primarily from the USA, Ukraine, and Russia. Only in Egypt, maize production was estimated at 7.50 million tons in 2020 (Al-Saidi, 2023). However, to meet the domestic demand, the country imported an additional 8.51 million tons. In the context of maize cultivation, both *M. incognita* and *M. javanica* nematodes were found in maize roots. The population density of *M. incognita* averaged 160 J2s per 250 grams of soil, whereas *M. javanica* had a higher density, averaging 1707 J2s per 250 grams of soil in the Alexandria and El-Behera governorates (**Figure 3**) (Ibrahim and Mokbel, 2009).

#### 3.1.2 The citrus industry is threatened by *Tylenchulus semipenetrans*

Citrus plays a crucial and strategic role in the national economies of North African countries. The citrus nematode *T. semipenetrans* affects citrus, grapes, and olive trees, leading to a gradual decline and crop loss (Duncan, 2005). Citrus production reached approximately 1.7 million metric tonnes each in Egypt and Algeria. In comparison, it reached 410,000 and 900,000 metric tonnes in Tunisia and Morocco, respectively, in 2022, as reported by the Food and Agriculture Organization (FAO, 2021). In the Asian part of the MENA region, before the crisis, Syria was producing 1 million tonnes of citrus annually (FAO, 2015). Moreover, citrus production extends to Saudi Arabia, where Najran and Alula regions contributed to the local market consumption by producing 176,687.2 tonnes in 2021 (World Data Atlas, 2021). Recently, in citrus orchards in Egypt, females ranged from 286-445 per gram of roots, while J2s and males were estimated at 1279- 3326 per 150 cm3 of soil (Abd-Elgawad et al., 2016). The loss of fruiting yield in Banzaheer lemon (*Citrus aurantifolia*) was estimated at 18% when the number was estimated with only 110 J2s per 150 cm3 soil; however, the loss escalated to 31% when the J2s number was 2300 J2s per 150 cm3 soil (Abd-Elgawad et al., 2016). In Morocco, the *T. semipenetrans* was the most predominant species, with 88% abundance frequency of the analyzed soil and root samples, with more than 1000 J2s per 100 grams of soil in regions like Beni Mellal-Khenifra and Berkane (Zoubi et al., 2022).

#### 3.1.3 *Heterodera avenae* is a threat to cereal yields

MENA countries import over 40% of the world’s wheat production (Bland, 2023). The total production of cereals (wheat, rice, and coarse grains) only in North Africa’s countries has been reduced to 33 million tonnes in 2023 compared to the last 5-year average, estimated at 37 million tonnes (FAO, 2023). Despite these challenges, the region has been making significant efforts to increase wheat production and self-sufficiency to meet the growing demand. The MENA region has experienced fluctuations in wheat production due to drought, pests, and political instability. In Saudia Arabia, according to the Saudi Grain Organization (SAGO), the domestic production of wheat is estimated at 600,000-700,000 metric tonnes in 2021/2022 and is subject to reach 1 million metric tonnes in 2022/2023 (USDA Foreign Agricultural Service, 2022). The attempts to expand production are constrained by nematodes and aggravate the shortage of cereal production. The prevalence of cereal cyst nematodes, particularly *Heterodera avenae*, was observed in cereal fields (Mokrini et al., 2012; Baklawa et al., 2015). For instance, in Egypt, the occurrence frequency of *H. avenae* varied from 3.7-28.6%, with an accompanying range of 75-1230 J2s and 7-35 cysts per 100 grams of soil, with a 21.6% reduction of grain yield (Korayem and Mohamed, 2015, 2018). These yield losses were even more severe in Saudi Arabia with 40-90% (Ibrahim et al., 1999) and 90% in Tunisia when soil analysis revealed 60 eggs/g soil (Namouchi-Kachouri et al., 2009).

#### 3.1.4 Olive nurseries and orchards face attacks from a diverse array of nematode species

When considering olive oil production specifically, the collective efforts of North African countries such as Algeria, Egypt, Libya, Morocco, and Tunisia result in a staggering production volume of 4.85 million tonnes (ICO, 2022). Furthermore, when considering the partial production of Palestine, Jordan, Syria, Saudi Arabia, and Iraq, the overall production exceeds 6 million tonnes (ICO, 2022). Meanwhile, plant-parasitic nematodes pose significant yield losses to olive orchards in the MENA region, mainly in the North African countries. Although these losses have not been estimated in the MENA region, the yield losses due to parasitic nematodes were between 22.3% and 28.6% in neighboring countries like Iran and Spain (Castillo et al., 2010; Sanei and Okhovvat, 2011). More than 100 diverse genera and species are related to olive cultivation in the MENA region with *Meloidogyne* spp., *Helicotylenchus* spp., *Pratylenchus* spp., *Xiphinema* spp., *Longidorus* spp., and *T. semipenetrans* among the most prevalent and damaging species (Castillo et al., 2010; Aït Hamza et al., 2017). The nematodes feed on olive tree roots and tissues, leading to stunted growth, tree death, and up to 50% reduction in fruit production (Abdel-Baset et al., 2022).

### 3.2 Agricultural practices leading to increased abundance and detrimental effects of nematodes

**Figure 4:**
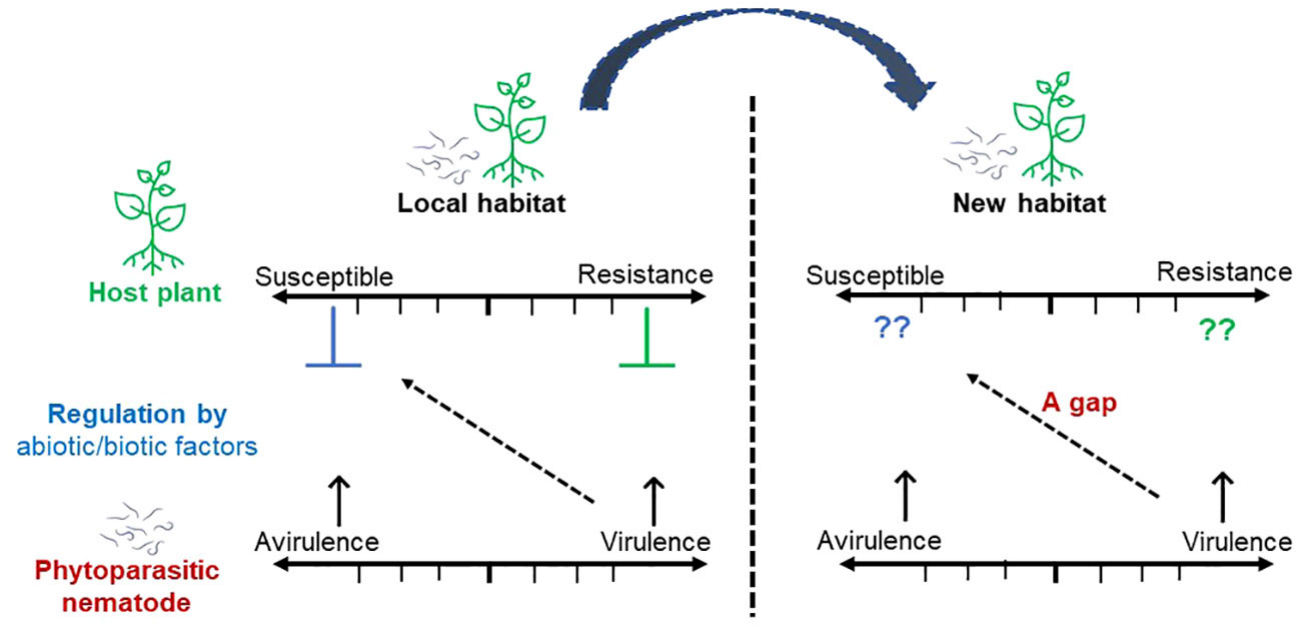
Schematic diagram illustrating the potential impact of imported plant species or genotypes and plant-parasitic nematodes invading a new habitat. The impact of plant-parasitic nematodes on crop yield is typically low in their native habitats due to regulatory abiotic and biotic factors. However, in new environments, host plants often become more susceptible, and nematode virulence can increase. This heightened impact may be due to 1) changes in abiotic factors like light exposure, 2) less effective natural antagonists, and 3) genetic changes in nematode populations due to selection, drift, or bottlenecks.

Soil transport poses risks to crops due to the spread of plant-parasitic nematodes by human activities and poor hygiene practices through infected plants (Bahadur, 2022). Before commercially available commercial inoculants, raw soil containing beneficial microbes like *Bradyrhizobium japonicum* was often transported to enhance nitrogen fixation in soybean fields. However, this practice inadvertently spread pests like soybean cyst nematodes (SCN), (*Heterodera glycine*) along with the beneficial microbes (Fairchild, 1948). Farmers in the MENA region, particularly farming in sandy desert soils, have historically attempted to restore barren land by transferring soil from established fields, believing such practices would enhance soil structure and biological activity. However, these practices contaminate the soil with parasites, pathogens, and dysfunctional microbial communities, causing significant disruptions to the overall ecological balance of the newly established system. Additionally, several plant-parasitic nematodes exhibit low levels of impact within their native ranges. However, they can have substantially more significant effects when introduced into new areas (Singh et al., 2013) (**Figure 4**). In the native habitat, the host plant-pathogen interaction is regulated by abiotic and biotic factors, determining their damage and impact on crop yield (Pandey et al., 2017). However, when the pathogen and host plant are transferred to or invaded in a new environment, the host plant likely becomes more susceptible (Velásquez et al., 2018). Pathogen virulence can increase due to the absence or lack of natural enemies or developing a paradigm shift from being an individual pathogen to shaping a disease complex or pathobiome context (Engering et al., 2013; Vayssier-Taussat et al., 2014; Godoy et al., 2023). This pronounced contrast in the host plant, or the nematode behavior, might be attributed to three different hypothetical scenarios: 1) Plant abiotic environmental factors, for example, the duration and intensity of light exposure, might influence host plant susceptibility. This could potentially result in enhanced susceptibility to nematode infections; 2) Antagonists could be less effective in suppressing the invasive nematode population; 3) The nematode population is likely exposed to events related to selection, genetic drift, or bottlenecks. These points should emphasize the potential impact of plant-parasitic nematodes in new environments and the need for appropriate management strategies to mitigate their spread and effect. Thus, the accidental spread of nematodes through the distribution of raw soil colonized with beneficial microbes highlights the importance of considering potential unintended ecological consequences when implementing agricultural practices. It also ensures the need for stringent hygienic measures to prevent the accidental dissemination of plant pathogens.

### 3.3 Lacking updated regulations and knowledge on various levels

The United States (US) and the European Union (EU) adopt eco-friendly measures to control nematodes, with the EU focusing on agricultural practices steering soil microbiomes (Elhady et al., 2018; Topalović et al., 2020; Elhady et al., 2021) and soil disinfection (Berger et al., 2022; Schumann et al., 2023). At the same time, the US emphasizes technology and genetically engineered crops (Sikora et al., 2023). For instance, in the USA, there is a recent emphasis on combining RNA interference (RNAi) with nanomaterials for targeted delivery to control nematodes (Opdensteinen et al., 2024). Conversely, these measurements are inadequate in the MENA region (Abd-Elgawad and Askary, 2015).

In cases where these policies already exist, the complexity of their implementation procedures is often regarded as problematic. Nevertheless, nematology-related infrastructure and training schemes must be more cohesive within each country. In Egypt, for instance, nematology is addressed via two main sectors: teaching, research, and extension, as well as universities and governmental agricultural research centers. These sectors mainly address basic and traditional research, but other significant issues need to be recognized among nematologists (Abd-Elgawad, 2019). Hence, we suggest the following themes (headlines for plant-parasitic nematode control) as inter-institutional research in the MENA region: 1) Characterizing plant-parasitic nematode biotypes and their engagement in management plans (Abd-Elgawad, 2021a), 2) Enforcing proper quarantine programs within the integrated nematode management, 3) Researching the biotic/abiotic factors for reliable and safe applications of biopesticides (Eida et al., 2018), 4) Use of emerging devices with high throughputs, such as biochemical markers and gene editing, as tools to expedite identification and development of nematode-resistant genotypes (Fullana et al., 2023), and 5) Extending determination and utility of nematode-damage thresholds, especially for economically important plant species/cultivars.

Furthermore, nematologists should utilize standardized procedures in nematode research, such as sampling, extraction, counting, and related practices (Abd-Elgawad, 2021b). Such standardization will allow future comparisons/reviews and may be followed up in comprehensive strategies with distinct points on the targeted goal(s) continuum. Current atypical sampling procedures may produce erratic results (Abd-Elgawad, 2021b). While functional sampling should be expanded, comparative sampling would enable a better understanding of the interactions between bio-control agent distribution and production practices to develop effective and safe IPM programs (Abd-Elgawad, 2022). These programs can maximize plant-parasitic nematode control and boost crop yields via dual-purpose, sequential, and co-application of production inputs in synergistic/additive ways that make them superior or complementary to chemical pesticides.

## 4 Discussion

**Figure 5:**
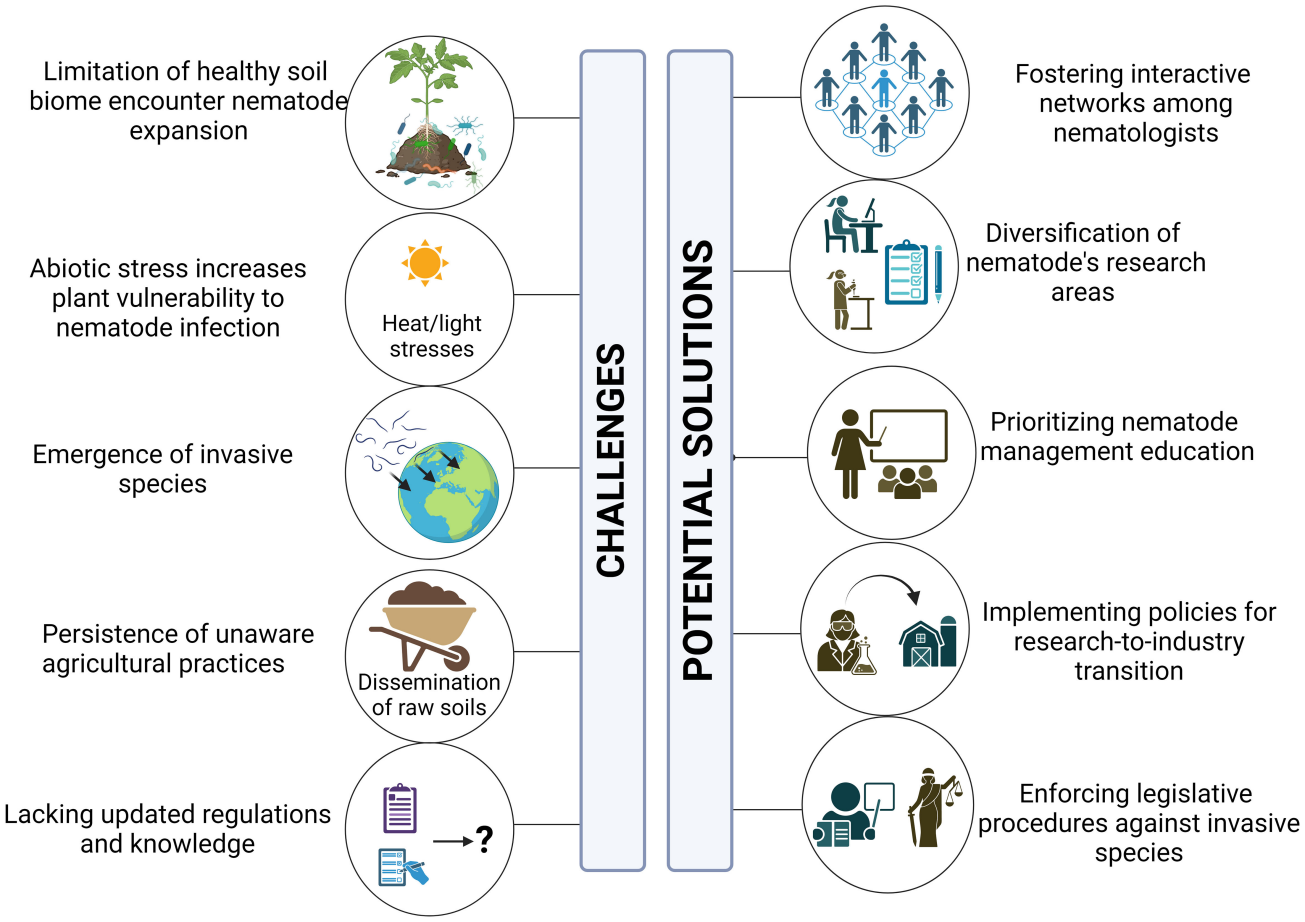
Challenges and potential solutions of plant-parasitic nematode in the arid desert landscape of the MENA region.

The MENA region is a disease-conducive part of the world, hosting a diverse array of plant-parasitic nematode genera, species, and genetically variable populations. Notably, *Meloidogyne* spp. frequently exceed damage threshold limits, posing significant risks to agricultural productivity. Other parasitic nematodes, including *T. semipenetrans*, *Rotylenchus* spp., *Ditylenchus* spp., and *Xiphinema* spp., present significant threats to agriculture industries such as citrus, olive, date palm, and other vegetable crops, with population densities frequently exceeding damage threshold limits. To raise awareness among farmers and agricultural cooperatives, as well as to better equip extension services, a multifaceted approach is proposed (**Figure 5**).

### 4.1 Interventions and actions to develop resilient management solutions

**Figure 6:**
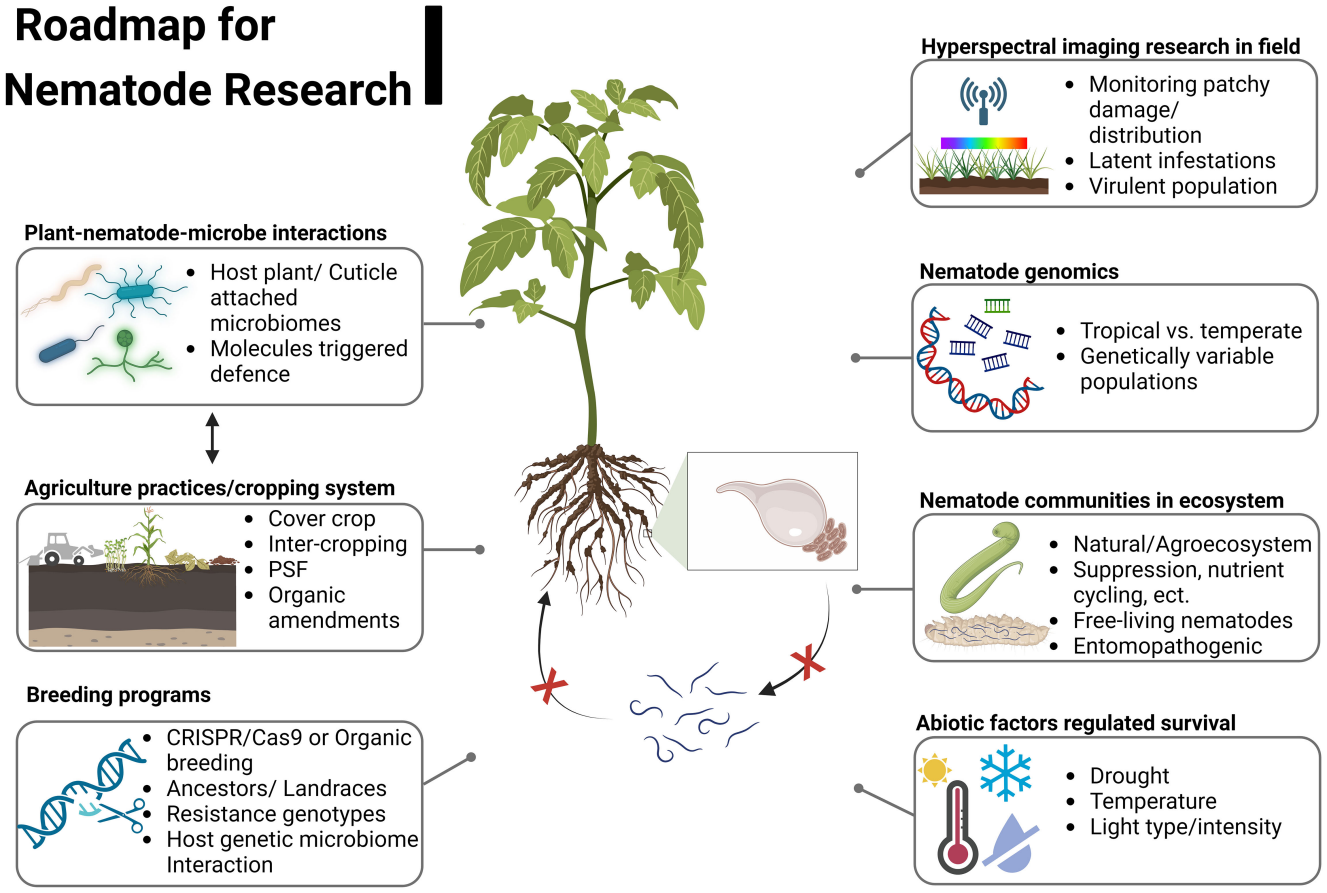
Areas of research that are urgently needed to accelerate the plant nematology field in the MENA region.

In the MENA region, efforts to find solutions to the problems of parasitic nematodes have mostly been individual efforts, which were limited to collecting samples and possibly inaccurate identification of the widespread species without finding resilient and sustainable solutions. Emphasis has sometimes been placed on recommending the utilization of internationally prohibited pesticides, leading to numerous complexities concerning human health and ecosystems. The nematicide market is predicted to grow from 2.10 billion USD in 2021 to 4.82 billion USD by 2026. Meanwhile, the bio-nematicide market was valued at only 220.0 million USD in 2021 and is projected to expand substantially at a CAGR of 15.0% from 2022 to 2028 [Markets and Markets™, INC, 2023]. The global orientation to replace bio- or microbial-based nematicides instead of synthetic chemicals will encounter a vast gap that has to be urgently filled. To link fundamental research to comprehend the underlying mechanisms governing nematode suppression with the development of eco-friendly products for plant-parasitic nematode management is an urgent need (**Figure 6**). Identifying nematode-suppressive endophytic bacterial strains or their metabolites and their mechanisms of action could lead to developing eco-friendly product(s) to fill the expected gap between banned chemicals and their expected bio-based nematicidal alternatives.

### 4.2 Rejuvenated practices for soil development in arid sandy soils

Cultivating crops can be challenging in arid regions characterized by sandy soils (Dou et al., 2023), often leading farmers to adopt ineffective and detrimental methods when restoring agricultural lands. Consequently, the transfer of raw soil from one field to another poses a significant risk for the dissemination of plant parasitic nematodes and other plant pathogens, especially in such vulnerable environments. However, recent practices have successfully improved the fertility and quality of sandy soils and supported sustainable farming in challenging arid environments. Among these practices, crop rotations, which include the initial cultivation of cover crops individually or in a mixture, can help to counteract soil compaction and revitalize nutrients and microbiomes (Agarwal et al., 2022). Cover crops such as legumes, grasses, brassicas, and composites can accelerate soil development and enhance organic matter levels (Mohamed et al., 2020). Some species can act as green manure through the “chop and drop” technique or as a forage for grazing, such as fenugreek (*Trigonella foenum-graecum* L.), grass pea (*Lathyrus sativus* L.), sweet-vetch (*Hedysarum boreale*), berseem clover (*Trifolium alexandrinum* L.), and sudangrass (*Sorghum drummondii*). Specific crops like cowpeas (*Vigna unguiculata* L.), sudangrass, and pearl millet (*Pennisetum glaucum* L.) are well-suited for cultivation in arid desert regions due to their ability to thrive under high temperatures and limited water availability. Remarkably, within a relatively short period of 2.5 months, these crops can yield substantial biomass ranging from 1.5 to 6 tons per acre, exhibiting an advantageous C/N ratio of 21-68, which indicates a good balance between carbon and nitrogen content for soil enrichment (Wang and Nolte, 2010). Summer legumes like cowpeas, lablab, and sesbania are crucial in arid regions due to their exceptional ability to fix nitrogen. They convert atmospheric nitrogen into a plant-usable form with 50 to 200 lbs per acre of fixed nitrogen (Wang and Nolte, 2010).

On the other hand, mulching soil surfaces with wood chips, straw, compost, or even terminating the cover crops can serve multiple functions that contribute to soil development quickly. Adding such layers to the soil surface can prevent erosion, enhance water infiltration, conserve moisture, and regulate temperature fluctuations (van Dung et al., 2022). This mulching might alleviate unfavorable traits of desert soils typically characterized by arid conditions, salinity, and poor soil quality. Also, adapted microbials, especially those endophytes isolated from extreme conditions, can be involved in agriculture practices like dripping irrigation, seed coating, nurseries, or foliar application (Eida et al., 2018). For example, plant residues or mulching materials require microbial decomposition, turning those materials into humus substances, a stable form of organic matter. Adding beneficial microbes, especially mycorrhiza and rhizobia, can contribute to soil nutrient cycling, facilitating plant nutrient uptake (Saad et al., 2020) and creating aggregates, enhancing soil aeration and water-holding capacity. Recently, other techniques such as solarization, nonclay, biochar, and humic acid application were proposed as fast-tech methods to develop desert soil for agriculture (Padidar et al., 2015; Alotaibi and Schoenau, 2019; Yi et al., 2022).

### 4.3 Expansion of nematode-related research areas

The plant-parasitic nematode research conducted in the MENA region requires coordination and needs to be more diverse. There is a pressing need to consolidate existing efforts and channel them towards finding sustainable solutions. We suggest the following research areas (**Figure 6**) that need to be established and extended beyond morphology-focused surveys boosting the delivery of effective and sustainable means of plant-parasitic nematode control.

#### 4.3.1 Plant-nematode-microbe interactions

Microbes and nematodes coexist in the soil, creating an essential microhabitat for plant health and ecosystem functioning. Identifying nematode-suppressive microbial strains, their metabolites, and mechanisms of action could lead to developing eco-friendly products to replace chemicals with bio-based nematicides.

#### 4.3.2 Crop system management for nematode control

Plants harbor a distinct microbiome in the rhizosphere that offers some defense against plant-parasitic nematodes. While the plant-soil feedback drives a functional trait-related legacy shift of the soil microbiome (Elhady et al., 2018), its impact on plant growth under the pressure of plant-parasitic nematodes remains unclear in the MENA region. Understanding plant-soil feedback by crop rotation, cover crop, or intercropping will contribute to exploring the role of microbial associations in nematode suppression and integrating the identified beneficial microbial associations in farming systems to increase crop productivity.

#### 4.3.3 Breeding programs harnessing plant-nematode-suppressing microbiomes

The continuous cultivation of resistant varieties often leads to the selection of nematode populations that can overcome the resistance of crops. In addition, the frequent domestication of crop genotypes might unintentionally lead to the disturbance of beneficial microbiomes due to a tradeoff with the targeted traits (Pérez-Jaramillo et al., 2018). Designing breeding programs based on better responsiveness and harnessing beneficial microbiomes through organic breeding or CRISPR/Cas9 genome editing might support the development of new crop genotypes that can be integrated into the sustainable management of nematode control.

#### 4.3.4 Hyperspectral imaging for detecting nematode infestation in crops

Hyperspectral imaging has recently been proposed for detecting diseases and plant parasitic nematode infestations in crops, even when combined with abiotic stresses (Žibrat et al., 2019). In arid climates, these infestations often coincide with drought, nutrient deficiencies, and salinity. Differentiating spectral signatures of nematode infestations from abiotic stresses requires advancing research in this area. Moreover, understanding the physiological and biochemical mechanisms behind these spectral responses is crucial for enhancing the hyperspectral imaging applications in agriculture, leading to more precise crop management under complex stress conditions.

#### 4.3.5 Genomics of tropical nematodes and genetic variability within populations

Exploring the functional genomics of tropical nematodes, especially in the MENA region, received little attention compared to temperate regions. The advances of high throughput next-generation sequencing shall provide more knowledge by revealing specific genes responsible for host interactions, parasitism regulation, and adaptations to harsh environments (Montarry et al., 2021). This research could shed light on nematode evolution, origin, and survival tactics across diverse environments, aiding targeted and sustainable nematode control. It also advances evolutionary biology studies, particularly in extreme environments. On the other hand, the high flexibility of plant-parasitic nematode genes associated with secreted effectors, suppression of host plant defense, and their tolerance to exposure to pesticides or bio-control agents are of great interest to understanding the mechanisms of their adaptation to antagonistic conditions, especially in modern agriculture. Understanding such mechanisms and related pathways should be the basis for developing diagnostic tools and targets for breeding durable nematode-resistant crop genotypes.

#### 4.3.6 Nematode communities in agriculture and natural ecosystems

Nematodes interact with other soil organisms like bacteria, fungi, and microarthropods, influencing their abundance and functions. Analyzing the functional structure of nematode taxa and species will help to understand their roles in regulating nutrient cycling and ecosystem functions. Their abundance, diversity, and community structure can also serve as crucial bioindicators of soil health and ecosystem quality as impacted by climate change and pollution.

#### 4.3.7 Entomopathogenic nematodes and endosymbiont bacteria

Agricultural production in the MENA region is confronted with a significant challenge by harmful insects and slug pests. Among these pests, the red palm weevil (*Rhynchophorus ferrugineus*), desert locust (*Schistocerca gregaria*), tomato leafminer (*Tuta absoluta*), and the recently emerging fall armyworm (*Spodoptera frugiperda*) are highly destructive, causing considerable economic losses. Entomopathogenic nematodes and their intimate symbiotic bacteria offer a promising means for combating many detrimental insects and slugs. While some research has been conducted in the MENA region, gaps exist in transitioning this research into practical applications and integration into farming systems.

#### 4.3.8 Abiotic factors regulating nematode survival and interactions with hosts and associated microbes

Abiotic factors, including extreme temperatures, limited water availability, salinity, and high levels of ultraviolet radiation, can significantly impact nematode infection and survival, as they can influence both the nematode and its host. The influence of abiotic factors on the survival of plant-parasitic nematodes, their dormancy patterns, and their interactions with host plants and associated microorganisms has been relatively unexplored in previous research of the MENA region. These factors are urgently needed as a priority research topic in the MENA region.

## 5 Conclusion: guidelines for future implementation

To address the pressing issues caused by plant-parasitic nematodes in the MENA region, a cohesive and interactive network of nematologists is crucial to fostering knowledge-sharing, research collaboration, and promoting sustainable practices. Linking basic research to the core mechanisms that control nematode suppression would help bridge the gap to practical products and practices. Collaboration with international partners such as the Julius Kuehn Institute, Ghent University, NemAfrica group in Kenya, Society of Nematologists in the USA, and the Organization of Nematologists of Tropical America (ONTA) can aid in technology transfer for eco-friendly pest control techniques. Nationally, collaborative efforts among educational institutions, research organizations, and policymakers are vital for raising awareness and providing consultancies to farmers. Besides, urgent legislative measures and international exchange plans are needed to prevent the spread of invasive species. Moreover, bridging the expected gap in replacing synthetic nematicide with bio products requires linking basic research to practical applications. Additionally, establishing specialized training programs and knowledge-sharing platforms, in collaboration with global nematology organizations, will enhance expertise and facilitate the exchange of best practices. Finally, policies must be implemented to develop farmland soils in arid regions to mitigate phytopathogen dissemination.

## Data availability statement

The datasets presented in this study can be found in online repositories. The names of the repository/repositories and accession number(s) can be found in the article/**Supplementary Material**.

## Author contributions

AE: Conceptualization, Data curation, Formal analysis, Investigation, Methodology, Project administration, Software, Validation, Visualization, Writing – original draft, Writing – review & editing. LA: Writing – review & editing. MA-E: Investigation, Writing – review & editing. HoH: Writing – review & editing. MS: Project administration, Writing – review & editing. HeH: Project administration, Supervision, Writing – review & editing.
