# Supplementary material for: Plant-parasitic nematode research in the arid desert landscape: a systematic review of challenges and bridging interventions
Source: Front Plant Sci. 2024 Jul 22;15:1432311. doi: 10.3389/fpls.2024.1432311 (PMC11298362; doi:10.3389/fpls.2024.1432311)
Supplement: Supplementary file 1 [file Table_1.pdf]

Supplementary Material

1 Supplementary Figures and Tables

Table S1: The population density (PD) and frequency of occurrence (%FO) ranges and associated damaged crops of the most economically important plant-parasitic nematodes in the studies undertaken within the MENA region over the past three decades. Values were normalized to (J2s or/and adults)/100cm^3 soil.

Country	City	Genus/Species	FO% range (J2s or/and adults)	PD range (J2s or/and adults)	PD Low/100 g	PD High/100 g	Soil sample size	Host plant	References
KSA	Jizan	*M. arenaria*	16-30.6	211-223	84.4	89.2	250 cm3	fruit trees	(Mokbel 2014)
Egypt	El-Nubaria district, El-Behera	*M. arenaria*	100	260.8-301.7	104.3	120.7	J2 (250 g soil)	Faba Bean	(Hammam et al. 2023)
Egypt	El-Nubaria district, El-Behera	*M. arenaria*	100	17.8-23.3	8.9		Egg masses (plant)	Faba Bean	(Hammam et al. 2023)
KSA	Jizan	*M. incognita*	28-38.7	215-231	86.0	92.4	250 cm3	fruit trees	(Mokbel 2014)
KSA	Jizan	*M. incognita*	29.4-37.9	197-215	78.8	86.0	250 cm3	Horticulture and ornamental plants	(Mokbel 2014)
Oman	Dhofar	*M. incognita*	30	60-2000	24.0	800.0	250 cm3 soil	Vegetables, filed crops, and fruit trees	(Mani et al. 1998)
Oman	Dhofar	*M. incognita*	18	100-5280	40.0	2112.0	250 cm3 soil	Vegetables, filed crops, and fruit trees	(Mani et al. 1998)
Egypt	Alexandria governorate	*M. incognita*	46-60	274-350	109.6	140.0	250 cm3 soil	lantana, spearmint, guava and olive trees	(Ibrahim and Handoo 2016)
Egypt	Upper Egypt	*M. incognita*	60-73	8.0-33.9	3.2	13.6	250g soil	Soybean	(Salem et al. 1994)
KSA	Jizan	*M. javanica*	30.6-32	219-234	87.6	93.6	250 cm3	fruit trees	(Mokbel 2014)
Oman		*M. javanica*	14.1	20-4000	8.0	1600.0	250 cm3 soil	Date palm	(Mani et al. 2005)
Oman	Batinah, Dhahira, Dhofar	*M. javanica*	11.83	20-1320	8.0	528.0	250 cm3 soil	Alfalfa	(Mani and Al Hinai 1996)
Iraq	Duhok, Kurdistan Region	*M. javanica*	3.47-80.5	337.5-1762	168.8	881.0	200g soil	Greenhouse's cucumber	(Ami et al. 2018)
Egypt	Upper Egypt	*M. javanica*	64-92	8.7-171.3	3.5	68.5	250g soil	Soybean	(Salem et al. 1994)
KSA	Riyadh	*Meloidogyne* spp.	45.8	306.4-3020.9	153.2	1510.5	200 cm3 soil	vegetable crops	(Almohithef et al. 2020)

KSA	Jizan	*Meloidogyne* spp.	30.6-32.3	192-209	76.8	83.6	250 cm3	Corn, Sesame, Sorghum	(Mokbel 2014)
KSA	Taif	*Meloidogyne* spp.	30	1630-6300	652.0	2520.0	250 gm soil	Rose	(Nour El-Deen et al. 2015)
Oman		*Meloidogyne* spp.	16.2	20-1500	8.0	600.0	250 cm3 soil	Date palm	(Mani et al. 2005)
Oman	Batinah, Dhahira, Dhofar	*Meloidogyne* spp.	14.2	20-550	8.0	220.0	250 cm3 soil	Alfalfa	(Mani and Al Hinai 1996)
Egypt	North Sinai	*Meloidogyne* spp.	27.3-48.1	20-3000	8.0	1200.0	250 cm3 soil	vegetables, field crops, fruit trees, ornamental and weed	(Korayem and Mohamed 2015)
Egypt	SEKEM organic farm	*Meloidogyne* spp.	3.75-100	10.0-17030	5.0	8515.0	200g soil	vegetables,fruit trees, and herbs	(Adam et al. 2013)
Egypt	Minufiya	*Meloidogyne* spp.	66.66-100	33-300	16.5	150.0	200g soil	Vegetable crops	(Bakr et al. 2011)
Egypt	Beheira	*Meloidogyne* spp.	100	66-810	33.0	405.0	200g soil	Vegetable crops	(Bakr et al. 2011)
Egypt	Sharkiya	*Meloidogyne* spp.	100	33-566	16.5	283.0	200g soil	Vegetable crops	(Bakr et al. 2011)
Egypt	North Eastern	*Meloidogyne* spp.	15-100	100-800	40.0	320.0	250g soil	Fruit Trees	(Abdel-Baset et al. 2022)
Egypt	North Eastern	*Meloidogyne* spp.	60-100	100-720	40.0	288.0	250g soil	Vegetable crops	(Abdel-Baset et al. 2022)
Egypt	Alexandria governorate	*Meloidogyne* spp.	56	340	136.0		250 cm3 soil	Sugar beet	(Ibrahim and Handoo 2016)
Egypt	Dakahlia	*Meloidogyne* spp.	46.3-81.3	32.8-66	13.1	26.4	250g soil	Potato	(Gad et al. 2018)
Sudan	Adu Hamad	*Meloidogyne* spp.	15.25-21.82	400–1200	400.0	1200.0	100g soil	Chickpea	(Mudawi et al. 2018)
KSA	Riyadh	*Ditylenchus* spp.	10.3	17.5- 97	8.8	48.5	200 cm3 soil	vegetable crops	(Almohithef et al. 2020)
KSA	Jizan	*Ditylenchus* spp.	9.7	39	15.6		250 cm3	fruit trees	(Mokbel 2014)
KSA	Jizan	*Ditylenchus* spp.	10-11.1	32-40	12.8	16.0	250 cm3	Horticulture and ornamental plants	(Mokbel 2014)
Egypt	North Sinai	*Ditylenchus* spp.	5.0-9.7	12.0-154	4.8	61.6	250 cm3 soil	vegetables, field crops, fruit trees, ornamental and weed	(Korayem and Mohamed 2015)
Egypt	SEKEM organic farm	*Ditylenchus* spp.	11.1-66.6	12.0-90	6.0	45.0	200g soil	vegetables, fruit trees, and herbs	(Adam et al. 2013)
Egypt	Alexandria governorate	*Ditylenchus* spp.	15	84	33.6		250 cm3 soil	Sugar beet	(Ibrahim and Handoo 2016)
Egypt	Alexandria governorate	*Ditylenchus* spp.	22.0-25	74-250	29.6	100.0	250 cm3 soil	lantana, spearmint, guava and olive trees	(Ibrahim and Handoo 2016)
Sudan	Adu Hamad	*Ditylenchus dipsaci*	12.73-15.25	200–1201	200.0	1201.0	100g soil	Chickpea	(Mudawi et al. 2018)
KSA	Jizan	*Helicotylenchus* spp.	12-16.1	62-78	24.8	31.2	250 cm3	Corn, Sesame, Sorghum	(Mokbel 2014)

KSA	Jizan	*Helicotylenchus* spp.	12.9-15.4	57-89	22.8	35.6	250 cm3	fruit trees	(Mokbel 2014)
KSA	Jizan	*Helicotylenchus* spp.	14.3-15.8	46-59	18.4	23.6	250 cm3	Horticulture and ornamental plants	(Mokbel 2014)
Oman	Batinah, Sharqia, Dhahira, Musandam	*Helicotylenchus* spp.	36.4	20-2700	8.0	1080.0	250 cm3 soil	Date palm	(Mani et al. 2005)
Oman	Batinah, Dhahira, Dhofar	*Helicotylenchus* spp.	21.89	15-960	6.0	384.0	250 cm3 soil	Alfalfa	(Mani and Al Hinai 1996)
Oman	Dhofar	*Helicotylenchus multicinctus*	31	20-2060	8.0	824.0	250 cm3 soil	Vegetables, filed crops, and fruit trees	(Mani et al. 1998)
Oman	Dhofar	*Helicotylenchus* spp.	18	30-360	12.0	144.0	250 cm3 soil	Vegetables, filed crops, and fruit trees	(Mani et al. 1998)
Egypt	North Sinai	*Helicotylenchus* spp.	5.0-11.1	40.0-180	16.0	72.0	250 cm3 soil	vegetables, field crops, fruit trees, ornamental and weed	(Korayem and Mohamed 2015)
Egypt	SEKEM organic farm	*Helicotylenchus* spp.	2.8-100	10.0-2300	5.0	1150.0	200g soil	vegetables, fruit trees, and herbs	(Adam et al. 2013)
Egypt	North Eastern	*Helicotylenchus* spp.	2.0-24.0	40-180	16.0	72.0	250g soil	Fruit Trees	(Abdel-Baset et al. 2022)
Egypt	North Eastern	*Helicotylenchus* spp.	10	120	48.0		250g soil	Wheat	(Abdel-Baset and Dawabah 2020)
Egypt	Alexandria governorate	*Helicotylenchus* spp.	42	172	68.8		250 cm3 soil	Sugar beet	(Ibrahim and Handoo 2016)
Egypt	Alexandria governorate	*Helicotylenchus pseudorobustus*	14.0-74	176-210	70.4	84.0	250 cm3 soil	lantana, spearmint, guava and olive trees	(Ibrahim and Handoo 2016)
Egypt	El-Giza	*Helicotylenchus indicus*	33	180	72.0		250 cm3 soil	Lantana camara L.	(Ibrahim and Handoo 2016)
Egypt	El-Giza	*Helicotylenchus pseudorobustus*	38	190	76.0		250 cm3 soil	Lantana camara L.	(Ibrahim and Handoo 2016)
Egypt	Upper Egypt	*Helicotylenchus pseudorobustus*	40-96	6.5-85.6	2.6	34.2	250g soil	Soybean	(Salem et al. 1994)
Moroco	Souss-Massa,Marrackech-Safi,Beni Mellal-Khenifra, Gharb,Berkane	*Helicotylenchus* spp.	75	3.0-14	3.0	14.0	100g soil	Citrus	(Zoubi et al. 2022)
KSA	Jizan	*Heterodera* spp.	11.5-12.5	65-74	26.0	29.6	250 cm3	Corn, Sesame, Sorghum	(Mokbel 2014)
Egypt	North Eastern	*Heterodera avenae*	8	80	32.0		250g soil	Wheat	(Abdel-Baset and Dawabah 2020)
Egypt	Ismailia	*Heterodera avenae*	79-92	13.9-45.6	7.0		Cysts (100 cm3 soil)	Wheat	(Baklawa et al. 2015)
Egypt	Ismailia	*Heterodera avenae*	70-91	6-13.5	6.0	13.5	Juveniles (100 cm3 soil)	Wheat	(Baklawa et al. 2015)

Egypt	Alexandria governorate	*Heterodera schachtii*	52	250	100.0		250 cm3 soil	Sugar beet	(Ibrahim and Handoo 2016)
Egypt	Alexandria	*Heterodera trifolii*	42	248	99.2		J2 250 cm3 soil	Clover	(Ibrahim et al. 2017)
Egypt	Alexandria, El-Behera	*Heterodera zeae*	42-60	310-362	124.0	144.8	J2 250 cm3 soil	Corn, Wheat	(Ibrahim et al. 2017)
Egypt	Alexandria, El-Behera	*Heterodera schachtii*	25.0-55	272-286	108.8	114.4	J2 250 cm3 soil	Sugar beet, Cabbage	(Ibrahim et al. 2017)
Egypt	Alexandria, Sohg	*Heterodera lespedezae*	40-42	232-260	92.8	104.0	J2 250 cm3 soil	Clover, Lentil	(Ibrahim et al. 2017)
Egypt	El-Behera	*Globodera rostochiensis*	44	284	113.6	0.0	J2 250 cm3 soil	Potato	(Ibrahim et al. 2017)
Egypt	El-Behera	*Heterodera avenae*	50	248	99.2	0.0	J2 250 cm3 soil	Wheat	(Ibrahim et al. 2017)
Egypt	Ismailia	*Heterodera avenae*	3.7-28.6	75-1230	75.0	1230.0	J2/ 100g soil	Wheat	(Korayem and Mohamed 2018)
Egypt	Ismailia	*Heterodera avenae*	3.7-28.6	3.0-23			White females in 1 g roots	Wheat	(Korayem and Mohamed 2018)
Egypt	Ismailia	*Heterodera avenae*	3.7-28.6	7.0-35			Cysts in l00 g soil	Wheat	(Korayem and Mohamed 2018)
Egypt	Upper Egypt	*Heterodera* spp.	40-64	6.3-59.9	2.5	24.0	250g soil	Soybean	(Salem et al. 1994)
Sudan	Adu Hamad	*Heterodera* spp.	5.63-18.18	200–1200	200.0	1200.0	100g soil	Chickpea	(Mudawi et al. 2018)
KSA	Jizan	*Pratylenchus* spp.	13.8-18.2	59-76	23.6	30.4	250 cm3	fruit trees	(Mokbel 2014)
KSA	Jizan	*Pratylenchus* spp.	15.8-17.6	55-72	22.0	28.8	250 cm3	Horticulture and ornamental plants	(Mokbel 2014)
KSA	Taif	*Pratylenchus* spp.	11.3	200-710	80.0	284.0	250 gm soil	Rose	(Nour El-Deen et al. 2015)
Oman		*Pratylenchus* spp.	25.3	20-340	8.0	136.0	250 cm3 soil	Date palm	(Mani et al. 2005)
Oman		*P. jordanensis*	50	157	62.8		250 cm3 soil	Major crops	(Mani et al. 2005)
Oman		*P. neglectus*	11	202	80.8		250 cm3 soil	Major crops	(Mani et al. 2005)
Oman	Batinah, Dhahira, Dhofar	*P. jordanensis*	38.46	20-1254	8.0	501.6	250 cm3 soil	Alfalfa	(Mani and Al Hinai 1996)
Oman	Dhofar	*P. jordanensis*	15	20-500	8.0	200.0	250 cm3 soil	Vegetables, filed crops, and fruit trees	(Mani et al. 1998)
Oman	Dhofar	*P. scribneri*	11	20-501	8.0	200.4	250 cm3 soil	Vegetables, filed crops, and fruit trees	(Mani et al. 1998)
Egypt	North Sinai	*Pratylenchus* spp.	5.0-33.0	8.0 - 23.0	3.2	9.2	250 cm3 soil	vegetables, field crops, fruit trees, ornamental and weed	(Korayem et al. 2014)
Egypt	SEKEM organic farm	*Pratylenchus* spp.	5.7-100	10.0-280	5.0	140.0	200g soil	vegetables, fruit trees, and herbs	(Adam et al. 2013)
Egypt	Alexandria governorate	*Pratylenchus* spp.	37	88	35.2		250 cm3 soil	Sugar beet	(Ibrahim and Handoo 2016)

Egypt	Alexandria governorate	*Pratylenchus* spp.	25.0-33	84-160	33.6	64.0	250 cm3 soil	lantana, spearmint, guava and olive trees	(Ibrahim and Handoo 2016)
Egypt	El-Giza	*P. thornei*	25	160	64.0		250 cm3 soil	Lantana camara L.	(Ibrahim and Handoo 2016)
Egypt	Upper Egypt	*P. zeae*	44-100	64-110	25.6	44.0	250g soil	Soybean	(Salem et al. 1994)
Egypt	Dakahlia	*Pratylenchus* spp.	25-47.1	11.1-99	4.4	39.6	250g soil	Potato	(Gad et al. 2018)
Moroco	Souss-Massa,Marrackech-Safi,Beni Mellal-Khenifra, Gharb,Berkane	*Pratylenchus* spp.	47	3.0-27	3.0	27.0	100g soil	Citrus	(Zoubi et al. 2022)
KSA	Taif	*Rotylenchus* spp.	29.3	850-2170	340.0	868.0	250 gm soil	Rose	(Nour El-Deen et al. 2015)
Oman		*R. anamictus*	13.1	400-12.600	160.0	5040.0	250 cm3 soil	Date palm	(Mani et al. 2005)
Oman		*Rotylenchus* spp.	15.2	40-960	16.0	384.0	250 cm3 soil	Date palm	(Mani et al. 2005)
Oman	Batinah, Dhahira, Dhofar	*Rotylenchulus reniformis*	15.98	20-4820	8.0	1928.0	250 cm3 soil	Alfalfa	(Mani and Al Hinai 1996)
Oman	Dhofar	*Rotylenchulus reniformis*	42	100-2800	40.0	1120.0	250 cm3 soil	Vegetables, filed crops, and fruit trees	(Mani et al. 1998)
Egypt	North Sinai	*Rotylenchulus reniformis*	5-12.7	8.0-1500	3.2	600.0	250 cm3 soil	vegetables, field crops, fruit trees, ornamental and weed	(Korayem et al. 2014)
Egypt	SEKEM organic farm	*Rotylenchus* spp.	12.5-100	10.0-2600	5.0	1300.0	200g soil	vegetables, fruit trees, and herbs	(Adam et al. 2013)
Egypt	North Eastern	*Rotylenchus* spp.	4.0-10	40-80	16.0	32.0	250g soil	Fruit Trees	(Abdel-Baset et al. 2022)
Egypt	North Eastern	*Rotylenchus* spp.	3.0-12	40-80	16.0	32.0	250g soil	Vegetable crops	(Abdel-Baset et al. 2022)
Egypt	Alexandria governorate	*Rotylenchulus reniformis*	16	94	37.6		250 cm3 soil	Sugar beet	(Ibrahim and Handoo 2016)
Egypt	Alexandria governorate	*Rotylenchus* spp.	12	86	34.4		250 cm3 soil	Sugar beet	(Ibrahim and Handoo 2016)
Egypt	Alexandria governorate	*Rotylenchulus reniformis*	17	86	34.4		250 cm3 soil	lantana, spearmint, guava and olive trees	(Ibrahim and Handoo 2016)
Egypt	El-Giza	*Rotylenchulus reniformis*	17	180	72.0		250 cm3 soil	Lantana camara L.	(Ibrahim and Handoo 2016)
Sudan	Adu Hamad	*Rotylenchulus reniformis*	1.91-16.36	200–1000	200.0	1000.0	100g soil	Chickpea	Mudawi et al.2018
KSA	Riyadh	*Tylenchorhynchus* spp.	12.9	97.1-477.2	48.6	238.6	200 cm3 soil	vegetable crops	(Almohithef et al. 2020)
KSA	Jizan	*Tylenchorhynchus mediterraneus*	29.36	99.91	40.0		250 cm3	Mango	(Mokbel 2014)

KSA	Jizan	*Tylenchorhynchus* spp.	20-22.5	134-146	53.6	58.4	250 cm3	Corn, Sesame, Sorghum	(Mokbel 2014)
KSA	Jizan	*Tylenchorhynchus* spp.	21.4-24	124-150	49.6	60.0	250 cm3	fruit trees	(Mokbel 2014)
KSA	Taif	*Tylenchorhynchus* spp.	9.8	220-860	88.0	344.0	250 gm soil	Rose	(Nour El-Deen et al. 2015)
Oman		*Tylenchorhynchus* spp.	17.2	10-130	4.0	52.0	250 cm3 soil	Date palm	(Mani et al. 2005)
Egypt	North Sinai	*Tylenchorhynchus* spp.	1.7-66.6	11.0-42.0	4.4	16.8	250 cm3 soil	vegetables, field crops, fruit trees, ornamental and weed	(Korayem and Mohamed 2015)
Egypt	SEKEM organic farm	*Tylenchorhynchus* spp.	5.7-62.5	12.0-450	6.0	225.0	200g soil	vegetables, fruit trees, and herbs	(Adam et al. 2013)
Egypt	North Eastern	*Tylenchorhynchus* spp.	5.0-50	60-180	24.0	72.0	250g soil	Fruit Trees	(Abdel-Baset et al. 2022)
Egypt	North Eastern	*Tylenchorhynchus* spp.	8.0-55	60-200	24.0	80.0	250g soil	Vegetable crops	(Abdel-Baset et al. 2022)
Egypt	North Eastern	*Tylenchorhynchus* spp.	14	180	72.0		250g soil	Wheat	(Abdel-Baset et al. 2022)
Egypt	Alexandria governorate	*Tylenchorhynchus* spp.	33	128	51.2		250 cm3 soil	Sugar beet	(Ibrahim and Handoo 2016)
Egypt	Alexandria governorate	*Tylenchorhynchus* spp.	28.0-40	76-112	30.4	44.8	250 cm3 soil	lantana, spearmint, guava and olive trees	(Ibrahim and Handoo 2016)
Egypt	North Sinai	*Tylenchus* spp.	22.2-33.3	27-42	10.8	16.8	250 cm3 soil	vegetables, field crops, fruit trees, ornamental and weed	(Korayem and Mohamed 2015)
Egypt	North Sinai	*Tylenchus* spp.	11.2-23.3	3.0-121.0	1.2	48.4	250 cm3 soil	vegetables, field crops, fruit trees, ornamental and weed	(Korayem et al. 2014)
Egypt	SEKEM organic farm	*Tylenchus* spp.	6.6-37.5	12.0-195	6.0	97.5	200g soil	vegetables, fruit trees, and herbs	(Adam et al. 2013)
Egypt	Alexandria governorate	*Tylenchus* spp.	17.0-18	56-96	22.4	38.4	250 cm3 soil	lantana, spearmint, guava, and olive trees	(Ibrahim and Handoo 2016)
Egypt	El-Giza	*Tylenchus* spp.	21	84	33.6		250 cm3 soil	Lantana camara L.	(Ibrahim and Handoo 2016)
Moroco	Souss-Massa,Marrackech-Safi,Beni Mellal-Khenifra, Gharb,Berkane	*Tylenchus* spp.	51	4.0-17	4.0	17.0	100g soil	Citrus	(Zoubi et al. 2022)
KSA	Jizan	*Trichodorus* spp.	14.68	82.22	32.9		250 cm3	Mango	(Mokbel 2014)
Egypt	Alexandria governorate	*Trichodorus* spp.	13	92	36.8		250 cm3 soil	Sugar beet	(Ibrahim and Handoo 2016)

KSA	Jizan	*Xiphinema elongatum*	11.01	50.79	20.3		250 cm3	Mango	(Mokbel 2014)
KSA	Jizan	*Xiphinema* spp.	17.2	72	28.8		250 cm3	Horticulture and ornamental plants	(Mokbel 2014)
KSA	Taif	*Xiphinema* spp.	16.5	740-2240	296.0	896.0	250 gm soil	Rose	(Nour El-Deen et al. 2015)
Egypt	North Sinai	*Xiphinema* spp.	8.9	10-983	4.0	393.2	250 cm3 soil	vegetables, field crops, fruit trees, ornamental and weed	(Korayem et al. 2014)
Egypt	SEKEM organic farm	*Xiphinema* spp.	8.3-20	18.0-180	9.0	90.0	200g soil	vegetables, fruit trees, and herbs	(Adam et al. 2013)
Egypt	North Eastern	*Xiphinema* spp.	3.0-14	20-120	8.0	48.0	250g soil	Fruit Trees	(Abdel-Baset et al. 2022)
Egypt	Alexandria governorate	*Xiphinema* spp.	16	86	34.4		250 cm3 soil	Sugar beet	(Ibrahim and Handoo 2016)
Egypt	Alexandria governorate	*Xiphinema* spp.	13	74	29.6		250 cm3 soil	lantana, spearmint, guava and olive trees	(Ibrahim and Handoo 2016)
Moroco	Souss-Massa,Marrackech-Safi,Beni Mellal-Khenifra, Gharb,Berkane	*Xiphinema* spp.	31	0-13	0.0	13.0	100g soil	Citrus	(Zoubi et al. 2022)
KSA	Riyadh	*Aphelenchoides* spp.	12	72- 80.3	36.0	40.2	200 cm3 soil	vegetable crops	(Almohithef et al. 2020)
KSA	Riyadh	*Aphelenchus* spp.	16.6	15.7- 87.3	7.9	43.7	200 cm3 soil	vegetable crops	(Almohithef et al. 2020)
KSA	Jizan	*Aphelenchus* spp.	16.51	61.64	24.7		250 cm3	Mango	(Mokbel 2014)
KSA	Jizan	*Hoplolaimus seinhorsti*	33.49	85.86	34.3		250 cm3	Mango	(Mokbel 2014)
Oman	Dhofar	*Rodopholus similis*	10	40-200	16.0	80.0	250 cm3 soil	Vegetables, filed crops, and fruit trees	(Mani et al. 1998)
Egypt	North Sinai	*Criconema* spp.	11.3-34.0	7.0- 40.0	2.8	16.0	250 cm3 soil	vegetables, field crops, fruit trees, ornamental and weed	(Korayem et al. 2014)
Egypt	SEKEM organic farm	*Hoplolaimus* spp.	6.6-33.3	10.0-550	5.0	275.0	200g soil	vegetables, fruit trees, and herbs	(Adam et al. 2013)
Egypt	North Eastern	*Criconema* spp.	10	80	32.0		250g soil	Fruit Trees	(Abdel-Baset et al. 2022)
Egypt	Alexandria governorate	*Aphelenchoides* spp.	14	143	57.2		250 cm3 soil	Sugar beet	(Ibrahim and Handoo 2016)
Egypt	Alexandria governorate	*Hoplolaimus* spp.	17	58	23.2		250 cm3 soil	Sugar beet	(Ibrahim and Handoo 2016)
Egypt	Alexandria governorate	*Mesocriconema* spp.	10	88	35.2		250 cm3 soil	Sugar beet	(Ibrahim and Handoo 2016)

Egypt	Alexandria governorate	*Aphelenchoides* spp.	18.0-21	75-152	30.0	60.8	250 cm3 soil	lantana, spearmint, guava and olive trees	(Ibrahim and Handoo 2016)
Egypt	Alexandria governorate	*Hoplolaimus* spp.	20	186	74.4		250 cm3 soil	lantana, spearmint, guava and olive trees	(Ibrahim and Handoo 2016)
Egypt	El-Giza	*Aglenchus geraerti*	25	120	48.0		250 cm3 soil	Lantana camara L.	(Ibrahim and Handoo 2016)
Egypt	El-Giza	*Bitylenchus ventrosignatus*	23	96	38.4		250 cm3 soil	Lantana camara L.	(Ibrahim and Handoo 2016)
Egypt	El-Giza	*Coslenchus capsici*	17	88	35.2		250 cm3 soil	Lantana camara L.	(Ibrahim and Handoo 2016)
Egypt	El-Giza	*Malenchus bryanti*	17	94	37.6		250 cm3 soil	Lantana camara L.	(Ibrahim and Handoo 2016)
Egypt	El-Giza	*Merlinius brevidens*	19	124	49.6		250 cm3 soil	Lantana camara L.	(Ibrahim and Handoo 2016)
Egypt	Upper Egypt	*Hoplolaimus seinhorsti*	40-86	6.8-22.3	2.7	8.9	250g soil	Soybean	(Salem et al. 1994)
Egypt	Upper Egypt	*Longidorus*	24-92	1.9-11.5	0.8	4.6	250g soil	Soybean	(Salem et al. 1994)
Egypt	Upper Egypt	*Merlinius brevidens*	92-100	25.9-74.7	10.4	29.9	250g soil	Soybean	(Salem et al. 1994)
Egypt	Dakahlia	*Longidorus*	2.9-23.8	24-51.5	9.6	20.6	250g soil	Potato	(Gad et al. 2018)
Sudan	Adu Hamad	*Aphelenchus avenae*	12.73-27.54	200–1800	200.0	1800.0	100g soil	Chickpea	(Mudawi et al. 2018)

Table S2: The reference of prevailing threshold limits associated with each genus or species used to assess the present status of nematode abundance within the MENA region compared to the available information in it or other regions. We know that the damage threshold limit is not a static parameter but is instead influenced by many variables; however, these limits were used as a standard to estimate the current situation of the most economically important nematodes and related problems in the MENA region. The threshold limit values were normalized to (J2s or/and adults)/100cm3 soil.

**Genus or species**	**Host plant or crop**	**Economic threshold/ normalized to (J2s or/and adults)/ 100cm^3 soil**	**References**
Meloidogyne spp.	Vegetables and ornamentals	10-100	(Todd and Jardine 1993)
M. arenaria	Potato	**415** (830 J2/200cm^3 soil)	(Korayem et al. 2012)
M. incognita	Watermelon	3.6	(Xing and Westphal 2012)
M. javanica	Fenugreek	130	(Nadeem et al. 2023)

Tylenchulus semipenetrans	Trufgrasse and, citrus	1600	(Bozbuga et al. 2023; Couch 1995; Nelson 1995)
Rotylenchulus spp.	Cotton	100	(Showmaker et al. 2011)
Helicotylenchus spp.	Grasses and cereals	400	(Fleming et al. 2016)
Xiphinema spp.	Trufgrasse	100	(Couch 1995)
Pratylechus spp.	Carrot	100	(Teklu et al. 2016)
Ditylenchus spp.	Onion	2	(Brinkman and Teklu 2021)
Tylenchorhynchus spp.	Trufgrasse	300	(Couch 1995; Nelson 1995)

Publication bibliography

Abdel-Baset, Sahar; Khalil, Ashraf; Mohamed, Shimaa (2022): Plant-Parasitic Nematodes Associated with Certain Fruit Trees and Vegetable Crops in the North Eastern Egypt. In *Egyptian Journal of Agronematology* 21 (2), pp. 110–121. DOI: 10.21608/ejaj.2022.267637.

Abdel-Baset, Sahar H.; Dawabah, Ahmed A. M. (2020): Phytonematodes Associating Wheat in North Eastern Egypt and Pathogenicity of Heterodera avenae on Certain Cereal Cultivars. In *Int. J. Phytopathol.* 9 (3), Article IJPP-3434, pp. 165–172. DOI: 10.33687/phytopath.009.03.3434.

Adam, Mohamed; Heuer, Holger; Ramadan, Elshahat M.; Hussein, M. A.; Hallmann, Johannes (2013): Occurrence of plant-parasitic nematodes in organic farming in Egypt. In *Int. J. Nematol. (International Journal of Nematology)* 23, pp. 82–90.

Almohithef, Abdallah H.; Al-Yahya, Fahad A.; Al-Hazmi, Ahmad S.; Dawabah, Ahmed A.M.; Lafi, Hamzeh A. (2020): Prevalence of plant-parasitic nematodes associated with certain greenhouse vegetable crops in Riyadh region, Saudi Arabia. In *Journal of the Saudi Society of Agricultural Sciences* 19 (1), pp. 22–25. DOI: 10.1016/j.jssas.2018.05.001.

Ami, Sulaiman Naif; Ghaeib, Shireen; Shingaly, Ali (2018): Disease incidence, identification, and monthly fluctuations in the population density of root-knot nematodes Meloidogyne javanica on cucumber plants in Semel District, Duhok, Kurdistan Region, Iraq. In *Acta Universitatis Sapientiae, Agriculture and Environment* 10 (1), pp. 52–65. DOI: 10.2478/ausae-2018-0005.

Baklawa, Mohamed; Niere, Björn; Heuer, Holger; Massoud, Samia (2015): Characterisation of cereal cyst nematodes in Egypt based on morphometrics, RFLP and rDNA-ITS sequence analyses. In *Nematol* 17 (1), pp. 103–115. DOI: 10.1163/15685411-00002855.

Bakr, R. A.; Mahdy, M. E.; Mousa, E. M. (2011): A survey of root-knot and citrus nematode in some newreclaimed land in Egypt. In *Pakistan Journal of Nematology* 29, pp. 165–170.

Bozbuga, R., Yildiz, S., Yuksel, E., Özer, G., Dababat, A. A. [A. A.], & İmren, M. (2023). Nematode-citrus plant interactions: Host preference, damage rate and molecular characterization of Citrus root nematode Tylenchulus semipenetrans. *Plant Biology (Stuttgart, Germany)*, *25*(6), 871–879. https://doi.org/10.1111/plb.13566

Brinkman, Pella; Teklu, Misghina Goitom (2021): Integrated nematode management of Ditylenchus dipsaci in onion: a nematode in a world all on its own. In Richard A. Sikora, Johan Desaeger, Leendert Molendijk (Eds.): Integrated nematode management: state-of-the-art and visions for the future. UK: CABI, pp. 297–303.

Couch, H. B. (1995): Diseases of Turfgrasses. In *Diseases of Turfgrasses*.

Fleming, T. R.; McGowan, N. E.; Maule, A. G.; Fleming, C. C. (2016): Prevalence and diversity of plant parasitic nematodes in Northern Ireland grassland and cereals, and the influence of soils and rainfall. In *Plant Pathol* 65 (9), pp. 1539–1550. DOI: 10.1111/ppa.12525.

Gad, S. B.; El Sherif, S. A.; Osman, M. A. (2018): Survey of Plant-Parasitic Nematode Genera Associated with Potato Plants at Dakahlia Governorate, Egypt. In *J Plant Pathol Microbiol* 09 (03), Article 1000436, pp. 1–4. DOI: 10.4172/2157-7471.1000436.

Hammam, Mostafa Mohamed Attia; Mohamed, Moawad Mohamed Mohamed; Abd-Elgawad, Mahfouz Mohamed Mostafa (2023): The Damage and Gain Threshold of Meloidogyne arenaria on Faba Bean Favors the Use of a Safe Biological Control Over Chemical Nematicides. In *PJN* 41 (1). DOI: 10.17582/journal.pjn/2023/41.1.8.17.

Ibrahim, I. K. A.; Handoo, Zafar (2016): Occurrence of phytoparasitic nematodes on some crop plants in northern Egypt. In *Pak. J. Nematol* 34 (02), pp. 163–169. DOI: 10.18681/pjn.v34.i02.p163.

Ibrahim, K. A.; Handoo, Zafar; Basyony, A. B. A. (2017): The cyst nematodes Heterodera and Globodera species in Egypt. In *Pak. J. Nematol* 35 (2), pp. 151–154. DOI: 10.18681/pjn.v35.i02.p151-154.

Korayem, A. M.; Mohamed, M. M. M. (2015): Damage Potential of Heterodera avenae on Wheat Growth and Yield in Relation to Nitrogen Fertilization in Egypt. In *Current Science International* 4 (4).

Korayem, A. M.; Mohamed, M.M.M. (2018): Occurrence and Geographical Distribution of Heterodera avenae on Some Cultivated Wheat Areas in Egypt. In *Egypt. J. Agronematol.* 17 (2), pp. 132–142.

Korayem, A. M.; Mohamed M M M; Hussein, Abou S. D. (2012): in Naturally and Artificially Infected Fields and its Effect on Some Tubers Properties. In *Journal of Applied Sciences Research* 8 (3), Article ISSN 1819-544X, pp. 1445–1452.

Korayem, A.M; Youssef, M.M.A; Mohamed, M.M.M; Lashein, A.M.S (2014): A Survey of Plant Parasitic Nematodes Associated with Different Plants in North Sinai. In *Middle East Journal of Agriculture Research* 3 (3), pp. 522–529.

Mani, A.; Al Hinai, M. S. (1996): Plant-parasitic nematodes associated with alfafa and fluctuations of Pratylenchus jordanensis population in the Sultanate of Oman. In *Fundam. appl. Nematol.* 20 (5), pp. 443–447.

Mani, A.; Al-Hinai, Muzna S.; Handoo, Zafar (1998): Plant-parasitic nematodes of crops in Dhofar Governorate Sultanate of Oman. In *Nematropica* 28 (1), pp. 61–69.

Mani, A.; Handoo, Zafar; Livingston, Sam (2005): Plant-parasitic nematodes associated with date palm trees (Phoenix dactylifera L.) in the Sultanate of Oman. In *Nematropica* 35 (2), pp. 135–144.

Mokbel, Asmaa A. (2014): Nematodes and their associated host plants cultivated in Jazan province, southwest Saudi Arabia. In *Egypt. J. Exp. Biol. (Zool.)* 10 (1), pp. 35–39.

Mudawi, Hanan Ibrahim; Idris, Mohamed Osman; Zawam, Hanaa Sedhum (2018): Occurrence and identity of the causal agents of wilt/root-rot disease in Chickpea in Abu Hamad area, Sudan. In *IJSR* 8 (1), pp. 1–14. DOI: 10.47556/J.IJSR.8.1.2018.1.

Nadeem, Hera; Khan, Amir; Gupta, Rishil; Anees, Arshi; Ahmad, Faheem (2023): A Seinhorst Model Determined the Host-Parasite Relationships of Meloidogyne Javanica Infecting Fenugreek Cv. UM202. In *Journal of Nematology* 55 (1), p. 20230005. DOI: 10.2478/jofnem-2023-0005.

Nelson, Eric B. (1995): Nematode Disorders of Turfgrasses: How Important are They? In *An independent newsletter for turf managers* 4 (10), pp. 1–20.

Nour El-Deen, A. H.; Darwesh, Hadeer Y.; El-Ghamdi, A. A.; Samra, B. N. (2015): Evaluating the Pathogenicity of Nematodes Infecting Roses at Taif Governorate, KSA. In *Research Journal of Pharmaceutical, Biological and Chemical Sciences (RJPBCS))* 6 (2), Article ISSN: 0975-8585, pp. 1562–1567.

Salem, A. A.; El-Morshedy, M.M.F.; El-Zawahry, A. M. (1994): Nematodes associated with soybean (Glycine max) in upper Egypt. In *Fundam. appl. Nematol.* 17 (5), pp. 401–404.

Showmaker, Kurt; Lawrence, Gary W.; Lu, Shien; Balbalian, Clarissa; Klink, Vincent P. (2011): Quantitative field testing Rotylenchulus reniformis DNA from metagenomic samples isolated directly from soil. In *PloS one* 6 (12), e28954. DOI: 10.1371/journal.pone.0028954.

Teklu, Misghina G.; Meressa, Beira H.; Radtke, Esther; Been, Thomas H.; Hallmann, Johannes (2016): Damage thresholds and population dynamics of Pratylenchus penetrans on carrot (Daucus carota L. cv. Nerac) at three different seed densities. In *Eur J Plant Pathol* 146 (1), pp. 117–127. DOI: 10.1007/s10658-016-0898-6.

Xing, Lijuan; Westphal, Andreas (2012): Predicting Damage of Meloidogyne incognita on Watermelon. In *Journal of Nematology* 44 (2), pp. 127–133.

Zoubi, Btissam; Mokrini, Fouad; Dababat, Abdelfattah A.; Amer, Mohammed; Ghoulam, Cherki; Lahlali, Rachid et al. (2022): Occurrence and Geographic Distribution of Plant-Parasitic Nematodes Associated with Citrus in Morocco and Their Interaction with Soil Patterns. In *Life (Basel, Switzerland)* 12 (5). DOI: 10.3390/life12050637.
